# ﻿A new psychrophilic yeast of Kriegeriaceae (Kriegeriales) isolated from lichen in the Arctic, with the description of *Licheniasvalbardensis* gen. et sp. nov.

**DOI:** 10.3897/mycokeys.114.135299

**Published:** 2025-02-24

**Authors:** Yukun Bai, Zeyu Tang, Xiaoya Peng, Jun Huang, Mingjing Sun, Jia Liu, Fang Peng

**Affiliations:** 1 China Center for Type Culture Collection (CCTCC), College of Life Sciences, Wuhan University, Wuhan, China Wuhan University Wuhan China; 2 Key Laboratory of Polar Environmental Monitoring and Public Governance, Ministry of Education of China, Beijing, China Ministry of Education of China Beijing China

**Keywords:** Kriegeriales, lichen, phylogeny, psychrophilic yeast, taxonomy

## Abstract

Yeasts are an important component of the microbiome in circumpolar regions that are characterized by unique environmental conditions. However, the taxonomy of yeasts remains largely unknown in high- and low-latitude regions. Curing a field survey of yeasts in the Svalbard Archipelago, Norway, a new yeast genus in Kriegeriales was isolated from dendritic lichens. Based on the phylogeny of multiple loci (ITS, LSU, SSU, *rpb1*, *rpb2*, *tef1-α*, and *cytb*), morphology, and physiological characteristics, the new genus *Lichenia* is proposed with the type species *Licheniasvalbardensis*. Additionally, 10 °C and 15 °C are the fastest growth temperatures of *L.svalbardensis*. It has low or no growth at temperatures above 20 °C, and there appears to be a morphogenetic transition from yeast to pseudohyphae or hyphae above 10 °C.

## ﻿Introduction

Basidiomycetous yeasts comprise decomposers, symbionts, and pathogens in different ecosystems (Buzzini and Martini 2000; Nagahama 2006; Buzzini et al. 2012; Peter et al. 2017; Sampaio and Gonçalves 2017; Sannino et al. 2017). Currently, five classes of Basidiomycota (Agaricostilbomycetes, Cystobasidiomycetes, Microbotryomycetes, Tremellomycetes, and Spiculogloeomycetes) are dominated by (dimorphic) species that comprise a yeast stage (Aime et al. 2006; Bauer et al. 2006; Hibbett et al. 2007; Boekhout et al. 2011; Weiß et al. 2014; Oberwinkler 2017; Li et al. 2020; Schoutteten et al. 2023). Microbotryomycetes, the second largest class in Pucciniomycotina (Basidiomycota), contains eight orders named Curvibasidiales, Heitmaniales, Heterogastridiales, Kriegeriales, Leucosporidiales, Microbotryales, Rosettozymales, and Sporidiobolales (Aime et al. 2006, 2014; Li et al. 2020; Schoutteten et al. 2023). In older classification systems, most of these species were lumped in artificial, large, polyphyletic genera such as *Sporobolomyces*, *Rhodotorula*, and *Tremella* (Li et al. 2020; Schoutteten et al. 2023; Jiang et al. 2024). With the use of molecular phylogenies as a base for yeast systematics, more than 2,000 species with yeast states have been proposed to accommodate the diversity of Basidiomycetous yeasts (Wang et al. 2015b; Li et al. 2020; Boekhout et al. 2022; Schoutteten et al. 2023). In the past, the placements of many monotypic genera in Microbotryomycetes were classified as incertae sedis (e.g., *Kriegeria*, *Meredithblackwellia*, *Pseudoleucosporidium*, *Psychromyces*, *Reniforma*, *Trigonosporomyces*, and *Udeniozyma*) (Aime et al. 2006; Wang et al. 2015a, 2015b; Schoutteten et al. 2023). The family Kriegeriaceae, identified with subgloboid spindle pole bodies and simple pore septa, was recognized by Toome et al. (2013) by using a phylogeny based on the SSU, LSU, and ITS regions of the ribosomal DNA. Toome et al. (2013) found that Kriegeriaceae have an interesting morphological feature: rosette-shaped budding patterns appear in culture conditions. Later, Wang et al. (2015b) reclassified the five *Rhodotorula* species in Kriegeriaceae into *Phenoliferia* spp. and *Yamadamyces* spp. Kriegeriaceae was not always recovered as a monophyletic lineage because of the contaminant protein-coding genes (*rpb1*, *rpb2*, *tef1-α*, and *cytb*) for the type strains of *Kriegeriaeriophori* (CBS 8387) and *Libkindiamasarykiana* (PYCC 6886) derived from *Candida* (Ascomycota) and the missing genes for *Meredithblackwelliaeburnea* (Schoutteten et al. 2023). Therefore, a robust molecular dataset that includes ITS, LSU, SSU, *rpb1*, *rpb2*, *tef1-α*, and *cytb* was important to clarify the phylogenetic position of the Kriegeriaceae and its internal relationships (Wang et al. 2015b; Masinova et al. 2017; Schoutteten et al. 2023).

Psychrophilic yeasts have been discovered in various groups of Basidiomycota, such as Cystobasidiomycetes, Microbotryomycetes, and Tremellomycetes (Margesin and Miteva 2011; Buzzini et al. 2012; Selbmann et al. 2014; Franca et al. 2016). Various species in Microbotryomycetes were described from polar regions. Perini et al. (2021) identified *Psychromycesglacialis* and *Camptobasidiumarcticum* from glaciers in Greenland and Svalbard. *Cryolevoniaschafbergensis*, a yeast unable to grow at 18 °C or higher temperatures, was collected from ancient permafrost and melted sea ice (Pontes et al. 2020). De Garcia et al. (2020) obtained two psychrophilic yeasts (*Cryolevoniagiraudoae* and *Camptobasidiumgelus*) from ice collected in cold environments. These psychrophilic yeast species in the genera *Camptobasidium*, *Glaciozyma*, *Cryolevonia*, and *Psychromyces* all cluster in Camptobasidiaceae (Schoutteten et al. 2023). Based on a phylogeny of ribosomal markers (ITS, LSU, and SSU), Toome et al. (2013) found that Camptobasidiaceae appeared as a sister lineage to Kriegeriaceae. In later analyses, the positions of Camptobasidiaceae and Kriegeriaceaea differed in phylograms based on the different datasets (protein-coding genes vs. ribosomal loci) (Wang et al. 2015a; Schoutteten et al. 2023). Currently, six genera, namely *Kriegeria*, *Kriegeriopsis*, *Libkindia*, *Meredithblackwellia*, *Phenoliferia*, and *Yamadamyces*, are recognized in Kriegeriaceae, and most of these were isolated from neotropical or temperate regions (Toome et al. 2013; Wang et al. 2015b; Masinova et al. 2017; Li et al. 2020; Diederich et al. 2022; Schoutteten et al. 2023). Species of *Kriegeria*, *Libkindia*, *Meredithblackwellia*, and *Yamadamyces* were isolated from neotropical or temperate forests in Asia, Europe, North America, or South America (Doubles and McLaughlin 1992; Golubev and Scorzetti 2010; Toome et al. 2013; Masinova et al. 2017; Li et al. 2020). *Kriegeriopsislivingstonensis* was described from Antarctica (Diederich et al. 2022). The remaining three *Phenoliferia* species were collected from glacier cryoconite, mud, and soil in Europe and identified as psychrophilic yeasts (Margesin et al. 2007). The family Camptobasidiaceae mainly comprises psychrophilic yeasts. Psychrophilic yeasts in Kriegeriaceae require further research.

Yeasts were isolated from numerous substrates, such as fruits, soil, insects, invertebrates, seawater, and wine (Nakase 2000; Whipps et al. 2008; Boekhout et al. 2022). However, yeasts related to lichen thalli remain largely unknown because lichens are substantially undersampled (Hawksworth and Grube 2020). Yeasts in Tremellomycetes, Cystobasidiomycetes, and Microbotryomycetes have been isolated from lichen in several studies (Cernajova and Skaloud 2019; Kachalkin et al. 2024; Schoutteten et al. 2024). *Lichenozymapisutiana* was isolated from *Cladonia* in Europe by Cernajova and Skaloud (2019) and was later reclassified to the genus *Occultifur* by Schoutteten et al. (2024). Nguyen et al. (2023) proposed *Microsporomycescladoniophilus* associated with the thalli of *Cladoniarei* in Japan. Based on a seven-loci phylogenetic reconstruction, Schoutteten et al. (2024) introduced the genus *Millanizyma* to accommodate this species. Various lichen-inhabiting yeasts in other genera (*Colacogloea*, *Cyrenella*, *Genolevuria*, *Teunia*, *Phaeotremella*, *Piskurozyma*, and *Piskurozyma*) were introduced by Kachalkin et al. (2024). However, the taxonomy of many yeast species associated with lichen lacked in Kriegeriaceae, especially in high-latitude regions.

Svalbard is located in a freezing area inside the Arctic Circle. It has an extremely cold and dry climate, with less than 10 °C of temperature and 500 mm precipitation annually (Forland et al. 2011). Various microorganisms have been investigated in this place. Singh and Singh (2012) reported the yeast and filamentous fungi from Svalbard and identified them as *Articulospora*, *Cryptococcus*, *Mrakia*, *Phialophora*, and *Rhodotorula*. In Svalbard, two ascomycetous yeasts (*Metschnikowiabicuspidata* and *M.zobellii*) were isolated from seawater and puddles on snow/ice (Butinar et al. 2011). Although some studies investigated the mycodiversity of these islands, limited knowledge is available about the diversity and taxonomy of yeast in this region. During the investigation of fungal diversity in Svalbard, Norway (78°13'12.91"N, 15°20'6.39"E), a piece of dendritic lichen was collected and a novel taxon was subsequently isolated. This study aims to reveal the taxonomy of this isolate combining the phylogenetic, physiological and morphological characteristics.

## ﻿Materials and methods

### ﻿Collection and isolation

During the survey of microbial diversity, specimens were collected in Longyearbyen, Svalbard, Norway, with the Chinese Arctic Scientific Expedition (applications to the Governor of Svalbard for research activity have been submitted in July 2014; RiS ID: 6754). Of which, a lichen in Usneaceae (might be *Usneasphacelata*) was collected. The whole lichen was sampled from the rock to a sterile envelope with a sterile blade. The lichen thallus was cut into small pieces and dissolved in sterile water. After grinding with magnetic beads for 15 min at 160 rpm, the microbial suspension was inoculated to plates containing different carbon sources media (cellulose, chitosan, petroleum, plastic, or xylose as the sole carbon sources). Emerging yeast colonies were transferred with a sterile bamboo skewer into a new potato dextrose agar media (PDA) plate. Plates were incubated at 10 °C for up to four weeks. Strains were deposited in the China Center for Type Culture Collection (CCTCC) and the Japan Collection of Microorganisms (JCM).

### ﻿DNA extraction and PCR amplification

After the strains were grown on PDA for four weeks, yeast cells were obtained for extraction of genomic DNA with the Plant/Fungus DNA Kit (Simgen, Hangzhou, China). Polymerase chain reactions (PCR) were conducted to amplify ITS, LSU, SSU, *rpb1*, *rpb2*, *tef1-α*, and *cytb*. The primers and PCR conditions are listed in Table 1. Purified PCR products were sequenced by Wuhan Nextomics Corporation (Wuhan, Hubei Province) using the PACBIO RS II platform. Consensus sequences were obtained from DNA sequences generated by each primer combination with the software Seqman v. 9.0.4 (DNASTAR Inc., Madison, WI, United States).

**Table 1.** Genes used in this study with PCR primers, primer DNA sequence, and optimal annealing temperature.

Locus	PCR primers	Amplification primers	PCR: thermal cycles: (Annealing temp. in bold)	Reference
ITS	ITS1	5’- TCCGTAGGTGAACCTGCGG -3’	(94 °C: 1 min, **52 °C**: 1 min, 72 °C: 1 min) × 35 cycles	White et al. 1990
	ITS4	5’- TCCTCCGCTTATTGATATGC -3’		
LSU	NL1	5’- GCATATCAATAAGCGGAGGAAAAG -3’	(94 °C: 1 min, **52 °C**: 1 min, 72 °C: 1 min) × 35 cycles	Kurtzman and Robnett 1998
	NL4	5’- GGTCCGTGTTTCAAGACGG -3’		
SSU	NS1	5’- GTAGTCATATGCTTGTCTC -3’	(94 °C: 1 min, **55 °C**: 30 s, 72 °C: 1.5 min) × 33 cycles	Sugita and Nakase 1999
	NS8	5’- TCCGCAGGTTCACCTACGGA -3’		
*rpb1*	RPB1-Af	5’- GARTGYCCDGGDCAYTTYGG -3’	(94 °C: 1 min, **52 °C**: 1 min, 72 °C: 1 min) × 35 cycles	Stiller and Hall 1997
	RPB1-Cr	5’- CCNGCDATNTCRTTRTCCATRTA -3’		
*rpb2*	fRPB2-5F	5’- GAYGAYMGWGATCAYTTYGG -3’	(94 °C: 30 s, **55 °C**: 30 s, 72 °C: 1 min) × 40 cycles	Liu et al. 1999
	fRPB2-7cR	5’- CCCATRGCTTGYTTRCCCAT -3’		
*tef1-α*	EF1-983F	5’- GCYCCYGGHCAYCGTGAYTTYAT -3’	(95 °C: 15 s, **50 °C**: 20 s, 72 °C: 1 min) × 35 cycles	Rehner and Buckley 2005
	EF1-1567R	5’- ACHGTRCCRATACCACCRATCTT -3’		
*cytb*	E1M4	5’- TGRGGWGCWACWGTTATTACTA -3’	(94 °C: 30 s, **49 °C**: 30 s, 72 °C: 2 min) × 35 cycles	Green et al. 2019
	E2 mr4	5’- AGCACGTARWAYWGCRTARWAHGG -3’		

### ﻿Morphological observation

To observe the morphological characters of the obtained yeasts, the strains were incubated in/on PDA (20% potato infusion, 2% glucose, 2% agar), PDB (20% potato infusion, 2% glucose), YM (0.3% yeast extract, 0.3% malt extract, 0.5% peptone, 1% glucose), or YMA (0.3% yeast extract, 0.3% malt extract, 0.5% peptone, 1% glucose, 2% agar) at 4 °C, 10 °C, 15 °C, and 20 °C for a month. The micromorphological features of the yeast cells were observed under an ICX41 microscope (Sunny Optical, Yuyao, China) at 1000× magnification. Over 30 yeast cells were measured to obtain the length and width. The cell culture characteristics (color, texture of colony) were recorded. To investigate the potential sexual cycles, the yeast cells were inoculated on CMA (5% corn meal infusion, 1.5% agar), MEA (5% malt extract, 2% agar), PDA, and YMA, according to Kurtzman et al. (2011). Yeast cells were incubated at 20 °C for one month.

### ﻿Phylogenetic analyses

The yeast isolate from the lichen was initially identified as Kriegeriaceae sp. based on the BLAST results in NCBI. A dataset of all currently known species in Kriegeriaceae and representative type species of other lineages in Microbotryomycetes was compiled based on recent published literature (Wang et al. 2015a, 2015b; Masinova et al. 2017; Li et al. 2020; Schoutteten et al. 2023, see Table 2). Contaminant sequences (*rpb1*, *rpb2*, *tef1-α*, and *cytb* for *Kriegeriaeriophori* and *Libkindiamasarykiana*) were removed from the dataset (Schoutteten et al. 2023). The compiled DNA sequence datasets of the different loci were aligned with the ClustalW algorithm in MEGA v. 6.0 (Tamura et al. 2013), after which the alignment was manually curated. The topologies between the different genetic loci were checked. The phylogenetic position of the newly discovered yeast was inferred through concatenating the alignments of the seven genetic regions (ITS, LSU, SSU, *rpb1*, *rpb2*, *tef1-α*, and *cytb*) to construct the phylogenetic tree. *Pseudomicrostromaphylloplana* (CBS 8073) and *Ustilagomaydis* (CBS 504.76) (Ustilaginomycotina, Basidiomycota) were used as the outgroup in the phylogenetic analyses. The maximum likelihood (ML) (Guindon et al. 2010) and Bayesian Inference (BI) analyses (Ronquist and Huelsenbeck 2003) were performed using PhyML v. 3.0 and MrBayes v. 3.1.2, respectively. FigTree v. 1.3.1 was used to show phylograms of Microbotryomycetes (Rambaut and Drummond 2010). The sequence data of *Licheniasvalbardensis* sp. nov. has been deposited in GenBank (Table 2). The concatenated seven-locus DNA sequence alignment used in this study has been deposited in TreeBASE (www.treebase.org; study ID 31855).

**Table 2.** Strains of Microbotryomycetes used in the molecular analyses in the present study.

Species	Strain	GenBank accession numbers						
		ITS	LSU	SSU	*rpb1*	*rpb2*	*tef1-α*	*cytb*
*Camptobasidiumarcticum*	EXF 12713H^T^	MN983248	MK454798	MT304813	NA	MT260386	MT260390	MT260394
*Camptobasidiumgelus*	EXF 12745^T^	AY040665	AY040647	NA	NA	NA	NA	NA
*Colacogloeafalcata*	JCM 6838^T^	AF444543	AF075490	AB021670	KJ708124	KJ708301	KJ707943	KJ707723
*Colacogloeafoliorum*	JCM 1696^T^	AF444633	AF317804	KJ708378	KJ708126	KJ708230	KJ707941	AB040622
*Colacogloeahydrangeae*	CGMCC 2.2798^T^	MK050451	NA	NA	MK849147	NA	MK849017	NA
*Colacogloearhododendri*	CGMCC 2.5821^T^	MK050452	NA	NA	MK849145	MK849286	MK849014	MK848887
*Curvibasidiumpallidicorallinum*	CBS 9091^T^	AF444641	AF444736	KJ708420	KJ708000	KJ708167	KJ707767	KJ707665
*Fellozymainositophila*	JCM 5654^T^	AF444559	AF189987	AB021673	KJ708136	KJ708306	KJ707951	KJ707718
*Glaciozymaantarctica*	JCM 9057^T^	AF444529	AF189906	DQ785788	KJ708131	KJ708182	NA	KJ707745
*Hamamotoalignophila*	CBS 7109^T^	AF444513	AF189943	KJ708372	KJ708139	KJ708241	KJ707953	KJ707637
*Hamamotoasingularis*	JCM 5356^T^	AF444600	AF189996	AB021690	KJ708140	KJ708336	KJ707957	KJ707716
*Kriegeriaeriophori*	CBS 8387^T^	AF444602	NR119455	DQ419918	NA	NA	NA	NA
*Kriegeriopsislivingstonensis*	AM1149^T^	ON922980	ON926889	NA	NA	NA	NA	NA
*Kriegeriopsislivingstonensis*	AM1150	ON922981	ON926890	NA	NA	NA	NA	NA
*Leucosporidiumcreatinivorum*	CBS 8620^T^	AF444629	AF189925	KJ708418	KJ708036	KJ708178	KJ707789	KJ707658
*Leucosporidiumfellii*	JCM 9887^T^	AF444508	AF189907	KJ708449	KJ708030	KJ708184	KJ707784	KJ707748
*Leucosporidiumfragarium*	CBS 6254^T^	AF444530	AF070428	KJ708413	KJ708031	KJ708179	KJ707791	AB040623
*Leucosporidiummuscorum*	CBS 6921^T^	AF444527	AF070433	KJ708414	KJ708038	KJ708180	KJ707793	AB040638
*Leucosporidiumscottii*	JCM 9052^T^	AF444495	AF070419	X53499	KJ708033	KJ708186	KJ707788	AB040658
*Leucosporidiumyakuticum*	CBS 8621^T^	AY212989	AY213001	KJ708419	NA	KJ708181	NA	KJ707659
*Libkindiamasarykiana*	PYCC 6886^T^	KU187885	KU187889	OP883947	NA	NA	NA	NA
***Licheniasvalbardensis***	**CCTCC AY 2022006^T^**	**OP866826**	**OP866960**	**OP866961**	**NA**	**OR485568**	**NA**	**NA**
***Licheniasvalbardensis***	**JCM 36172**	**PQ164714**	**PQ164717**	**PQ164721**	**NA**	**OR485569**	**NA**	**NA**
*Meredithblackwelliaeburnea*	CBS 12589^T^	JX508799	JX508798	JX508797	NA	NA	NA	NA
*Microbotryumviolaceum*	CBS 143.21^T^	KJ708462	KJ708462	KJ708388	KJ708042	KJ708192	KJ707811	KJ707613
*Microstromaphylloplanum*	CBS 8073^T^	AB038131	AF190004	AJ496258	KP322906	KP323063	KP323116	AB041051
*Oberwinklerozymadicranopteridis*	CGMCC 2.3441^T^	MK050426	NA	NA	MK849162	MK849300	NA	MK848901
*Oberwinklerozymanepetae*	CGMCC 2.5824^T^	MK050427	NA	NA	MK849254	MK849391	NA	MK848992
*Oberwinklerozymayarrowii*	JCM 8232^T^	AF444628	AF189971	AB032658	NA	KJ708275	KJ707938	KJ707735
*Phenoliferiaglacialis*	CBS 10436^T^	EF151249	EF151258	KJ708381	KJ708067	KJ708233	KJ707831	KJ707597
*Phenoliferiapsychrophenolica*	CBS 10438^T^	EF151246	EF151255	KJ708382	KJ708071	KJ708259	KJ707859	KJ707598
*Phenoliferiapsychrophila*	CBS 10440^T^	EF151243	EF151252	KJ708383	NA	KJ708260	KJ707833	KJ707599
*Pseudohyphozymabogoriensis*	JCM 1692^T^	AF444536	AF189923	KJ708363	KJ708130	KJ708216	KJ707949	AB040619
*Pseudohyphozymahydrangeae*	CGMCC 2.2796^T^	MK050443	NA	NA	MK849126	MK849287	MK849015	MK848888
*Pseudohyphozymalulangensis*	CGMCC 2.2612^T^	MK050442	NA	NA	MK849129	MK849270	NA	MK848875
*Pseudohyphozymapustula*	JCM 3934^T^	AF444531	AF189964	KJ708361	KJ708128	KJ708261	KJ707937	AB040642
*Psychromycesglacialis*	EXF 13111^T^	MK671633	MT301949	MT248408	NA	MW036268	MT260389	MT260392
*Rhodosporidiobolusazoricus*	JCM 11251^T^	AB073229	AF321977	AB073269	KJ708053	KJ708202	KJ707813	KJ707693
*Rhodosporidiobolusfluvialis*	JCM 10311^T^	AY015432	AF189915	AB073272	KJ708046	KJ708204	KJ707816	KJ707679
*Rhodosporidiobolusjianfalingensis*	CGMCC 2.3532^T^	MK050402	NA	NA	MK849179	MK849317	MK849048	MK848917
*Rhodosporidiobolusmicrosporus*	JCM 6882^T^	AF444535	AF070436	KJ708441	KJ708054	KJ708284	KJ707817	KJ707724
*Rhodosporidiobolusodoratus*	JCM 11641^T^	KJ778638	AF387125	KJ708427	KJ708045	KJ708322	KJ707819	KJ707694
*Rhodosporidiobolusruineniae*	JCM 1839^T^	AF444491	AF070434	AB021693	KJ708052	KJ708286	KJ707820	KJ707700
*Rhodotorulaaraucariae*	JCM 3770^T^	AF444510	AF070427	KJ708435	KJ708096	KJ708209	KJ707862	AB041048
*Rhodotorulababjevae*	JCM 9279^T^	AF444542	AF070420	AB073270	NA	NA	KJ707874	KJ707746
*Rhodotorulaglutinis*	JCM 8208^T^	AF444539	AF070429	X69853	NA	NA	KJ707869	AB040626
*Rhodotorulagraminis*	JCM 3775^T^	AF444505	AF070431	X83827	KJ708093	KJ708234	KJ707868	AB040628
*Slooffiacresolica*	JCM 10955^T^	AF444570	AF189926	KJ708365	KJ708135	KJ708222	KJ707942	NA
*Slooffiapilatii*	JCM 9036^T^	AF444598	AF189963	KJ708364	KJ708137	KJ708256	KJ707947	AB040641
*Sporobolomycesjohnsonii*	CBS 5470^T^	AY015431	AY015431	AY015431	AY015431	AY015431	AY015431	AY015431
*Ustilagomaydis*	CBS 504.76^T^	AF453938	AY854090	X62396	XM401478	AY485636	AY885160	AB040663
*Yamadamycesrosulatus*	CBS 10977^T^	EU872492	EU872490	KJ708384	KJ708083	KJ708263	KJ707854	KJ707607
*Yamadamycesterricola*	CGMCC 2.5820^T^	MK050425	NA	NA	MK849127	MK849268	MK848999	MK848874
*Yurkovialongicylindrica*	CGMCC 2.5603^T^	MK050441	NA	NA	MK849218	MK849357	MK849084	MK848952

^1^CBS: Westerdijk Fungal Biodiversity Institute (CBS-KNAW Fungal Biodiversity Centre), Utrecht, The Netherlands; CCTCC, China Center for Type Culture Collection, Wuhan, China; CGMCC, Chinese General Microbiological Culture Collection Center, Beijing, China; EXF, Microbial Culture Collection Ex of the Infrastructural Centre Mycosmo, Ljubljana, Slovenia; JCM, Japan Collection of Microorganisms, RIKEN BioResource Center, Saitama, Japan; PYCC, Portuguese Yeast Culture Collection, Caparica, Portugal; NA: not applicable. All the new isolates used in this study are in bold, and the type materials are marked with T.

### ﻿Biochemical and physiological tests

Biochemical and physiological tests were performed according to the protocols described by Kurtzman et al. (2011). All results were recorded 30 days post inoculation. The test tubes were sterilized by 1 N HCl to guarantee their cleanliness in assimilation tests. Starved cells were prepared through shaking in 1 mL of sterilized water for 7 days at 10 °C. For growth tests on carbon compounds, each tube of YNB medium containing carbon compound equal to 0.5% glucose was inoculated with starved cells, and YCB containing nitrogen compound equal to 0.0108% of nitrogen for nitrogen growth tests. Starved cells were inoculated on a vitamin-free yeast base in vitamin-free growth tests. Cell cultures were serially diluted 10/10^2^/10^3^/10^4^/10^5^-fold, spotted onto PDA medium, and incubated for 7 days to measure the growth at various temperatures (4 °C, 10 °C, 15 °C, 20 °C, 22.5 °C, 25 °C). Tolerance of NaCl was tested with 10% NaCl concentrations (10% NaCl, 5% glucose, 0.2% (NH4)_2_SO_4_, 0.02% MgSO_4_, 0.001% CaCl_2_, 0.00001% FeSO_4_, 0.15% Na_2_HPO_4_, 0.15% K_2_HPO_4_, and 2% agar). The growth in high osmotic pressure was measured in PDA plates with 50% D-Glucose. To measure the growth curve of *Licheniasvalbardensis* sp. nov., 300 μL plateau cells were inoculated to 30 mL PDB at 10 °C for 10 days. The values for optical density of yeast cells at 600 nm (OD600) were measured by using the spectrophotometer. For the hydrolysis test of urea, cells from PDA slant were incubated on Christensen’s urea agar slant (0.1% peptone, 0.5% NaCl, 0.2% (NH_4_)H_2_PO_4_, and 0.0012% phenol red, 2% agar) for four days. DBB reagent was applied to the surface of the culture to conduct the diazonium blue B color reaction. Three replicates were conducted for each test. The result of physiological tests has been recorded below.

## ﻿Result

### ﻿Phylogeny

**Figure 1.**
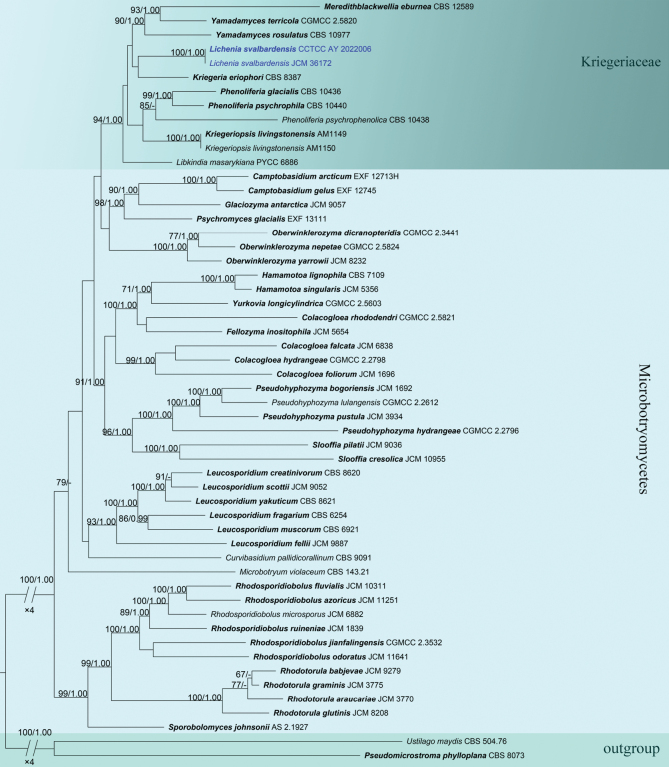
Phylogram of Microbotryomycetes resulting from a maximum likelihood analysis based on a combined matrix of ITS, LSU, SSU, *rpb1*, rpb2, *tef1-α*, and *cytb*. Numbers above the branches indicate ML bootstraps (left, ML BS ≥ 70%) and Bayesian Posterior Probabilities (right, BPP ≥ 0.95). The tree is rooted with *Pseudomicrostromaphylloplana*CBS 8073 and *Ustilagomaydis*CBS 504.76. Isolates from the present study are marked in blue, and holotype isolates are made in bold.

The phylogenetic position of *Licheniasvalbardensis* in Microbotryomycetes was analyzed based on two datasets, namely a concatenated seven-loci dataset (SSU, ITS, LSU, *rpb1*, *rpb2*, *tef1-α*, and *cytb*) and a concatenated ITS and LSU dataset. The seven loci analyses were similar to the tree topologies of the combined analyses. The dataset consisted of 54 isolates representing 52 species and 25 genera, including two outgroup taxa (*Pseudomicrostromaphylloplana*CBS 8073 and *Ustilagomaydis*CBS 504.76). The total length of the concatenated seven-locus alignment was 10,732 characters, including gaps (2,341 for SSU, 924 for ITS, 652 for LSU, 1,265 for *rpb1*, 1,722 for *rpb2*, 3,378 for *tef1-α*, and 430 for *cytb*), and 1,580 characters, including gaps (924 for ITS and 652 for LSU), for the ITS+LSU alignment. The phylogram of the concatenated dataset resulting from ML analyses was similar to the result of BI analyses. ML bootstraps (ML BS ≥ 70%) and Bayesian Posterior Probabilities (BPP ≥ 0.95) were given at the nodes in the phylograms. (Fig. 1, Suppl. material 1). The phylogenetic trees reveal that *L.svalbardensis* had a close relation with *Phenoliferia*, *Kriegeria*, *Kriegeriopsis*, *Libkindia*, *Meredithblackwellia*, and *Yamadamyces* with high support value (ML/BI = 94/1.00), which has been described below.

### ﻿Taxonomy

#### ﻿ Lichenia

Taxon classificationFungiKriegerialesKriegeriaceae

Zeyu Tang & Fang Peng gen. nov.

327A400D-9E1F-5124-93D7-79385D969F2D

846865

##### Etymology.

The name reflects the organism that the species was isolated from, lichen.

##### Type species.

*Licheniasvalbardensis* Zeyu Tang & Fang Peng

##### Culture characteristics.

Colonies on PDA butyrous, white. Hyphae, pseudohyphae, and budding cells were observed. Hyphae and pseudohyphae hyaline, unbranched, white to grey, septate. Cells and budding cells hyaline, ellipsoidal, smooth, guttulate. Sexual reproduction not known.

##### Notes.

In the phylogenetic trees, *Kriegeria*, *Kriegeriopsis*, *Libkindia*, *Lichenia*, *Meredithblackwellia*, *Phenoliferia*, and *Yamadamyces* were clustered in Kriegeriaceae (Fig. 1, Suppl. material 1). The identity rates of ITS and LSU between *Lichenia* and other genera in Kriegeriaceae are lower than the genera thresholds of 96.31% for ITS and 97.11% for LSU (Table 3), agreeing with the taxonomic thresholds predicted by Vu et al. (2016). Therefore, we propose *Lichenia* as a new genus in Kriegeriaceae.

**Table 3.** Identity rates in ITS and LSU between *Licheniasvalbardensis* and other species in Kriegeriaceae (%).

Species	ITS	LSU
***Kriegeriaeriophori***	88.36%	95.87%
***Kriegeriopsislivingstonensis***	86.60%	96.00%
***Libkindiamasarykiana***	93.71%	95.60%
***Meredithblackwelliaeburnea***	86.41%	91.60%
***Phenoliferiaglacialis***	90.36%	95.71%
***Phenoliferiapsychrophenolica***	89.38%	95.84%
***Phenoliferiapsychrophila***	89.80%	96.38%
***Yamadamycesrosulatus***	89.32%	96.05%
***Yamadamycesterricola***	89.44%	96.38%

#### ﻿ Lichenia svalbardensis

Taxon classificationFungiKriegerialesKriegeriaceae

Zeyu Tang & Fang Peng sp. nov.

3AE705FE-4662-5C5B-B729-5303D147C30E

846866

Fig. 2

##### Etymology.

The name reflects the station where this species was collected, Svalbard, Norway.

##### Specimens examined.

Norway, Svalbard, isolate from dendritic lichen (Usneaceae) on the rock, 78°13'12.91"N, 15°20'6.39"E, Jul. 2014, Fang Peng (holotype CCTCC AY 2022006, preserved in a metabolically inactive state; other living culture: JCM 36172).

##### Culture characteristics.

**Figure 2.**
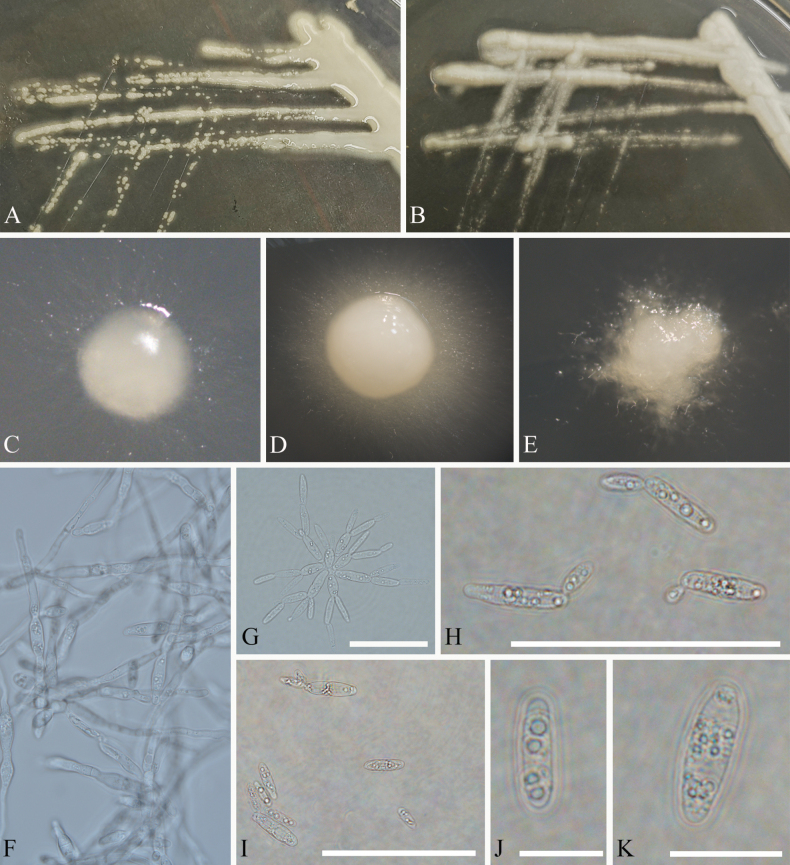
Morphology of *Licheniasvalbardensis***A–E** cultures after incubation for 1 week **A** cultures on YMA at 10 °C **B** cultures on YMA at 20 °C **C** single colony on YMA at 4 °C **D** single colony on YMA at 10 °C **E** single colony on YMA at 20 °C **F** hyphae **G** pseudohyphae **H** apically budding yeast cells **I–K** yeast cells. Scale bars: 50 µm (**G, I**); 30 µm (**H**); 10 µm (**J–K**).

On YMA and PDA plates, after 7 days and 30 days at 4 °C, cultures are smooth, butyrous, creamy-white, without hypha around the single colony (Fig. 2C); after 7 days and 30 days at 10 °C and 15 °C, cultures white to yellowish, smooth, butyrous, filamented margin, hyphae grow around the most single colony (Fig. 2D); after 7 days and 30 days at 20 °C, cultures white to yellowish, with rough surface and edge, smooth single colonies are observed seldomly (Fig. 2E).

##### Micromorphology.

In YM and PD broth, yeast cells are hyaline, ellipsoidal, smooth, guttulate, 9.5–15.6 × 3.4–4.5 µm (av. = 12.6 ± 3.5 × 4.0 ± 0.8 µm, n = 30), with a gelatinous sheath (Fig. 2I–K). Budding is enteroblastic and occurs on a narrow base from each pole (Fig. 2H). After 7 days at 10 °C, pseudohyphae are formed; at 15 °C and 20 °C, numerous pseudohyphae and hyphae are formed (Fig. 2F–G), numerous yeasts forming rosettes (Fig. 2G). Sexual structures are not observed on YMA, PDA, and CMA. Ballistoconidia are not produced.

##### Notes.

*Licheniasvalbardensis* was isolated from lichen in polar habitats. Numerous yeast cells of *Licheniasvalbardensis* clustered and formed rosettes. It is consistent with the morphological characteristics of Kriegeriaceae (Toome et al. 2013). In the seven loci phylogenetic analyses, *L.svalbardensis* from lichen (Usneaceae) formed a well-supported monophyletic clade, distinct from *Kriegeriaeriophori*, *Libkindiamasarykiana*, and *Meredithblackwelliaeburnea* (Fig. 1). Morphologically, cells of *L.svalbardensis* (9.5–15.6 × 3.4–4.5 µm) are shorter than *Meredithblackwelliaeburnea* (12.6–17.6 × 3.9–5.2 µm), wider than *Libkindiamasarykiana* (8.5–12.0 × 2.0–3.0 µm), and shorter than *Kriegeriaeriophori* (23.0–29.0 × 4.0–5.0 µm) (Doubles and McLaughlin 1992; Toome et al. 2013; Masinova et al. 2017). Therefore, we kept *L.svalbardensis* separate.

### ﻿Physiological and biochemical characteristics

Physiological characteristics of *Licheniasvalbardensis* in the current study have been measured. In detail, D-(+)-glucose, inulin, β-lactose, maltose, methyl-α-D-glucoside, D(+)-raffinose, sucrose, and D-(+)-xylose fermentation are negative. D-(+)-glucose, D-(+)-cellobiose, ethanol, D-(+)-galactose, D-gluconate, D-glucitol, β-lactose, L-(+)-arabinose, maltose, D-(+)-melibiose, D-(+)-melezitose, ribitol, D(+)-raffinose, L-rhamnose, D-(-)-ribose, D-(+)-trehalose, xylitol, citrate (weak), D-arabinose (weak), inulin (weak), DL-lactate (weak), D-mannitol (weak), D-glucosamine (delayed), and D-(+)-xylose (delayed) are assimilated as sole carbon sources. Meso-erythritol, glycerol, galactitol, myo-inositol, methyl-α-D-glucoside, L-(-)-sorbose, and sucrose are not assimilated. Ethylamine, N-acetyl-D-glucosamine, nitrate, nitrite, and creatinine (delayed) are assimilated as sole nitrogen sources. Cadaverine, D-glucosamine, and L-lysine are not assimilated. The maximum growth temperature is 20 °C. Growth in vitamin-free medium is positive. Growth on 50% (w/w) glucose yeast extract agar is negative. Growth on glucose agar with 10% NaCl is negative. Urease activity is positive. Diazonium blue B reaction is positive. Comparisons of physiological characteristics of *L.svalbardensis* and other members of Kriegeriaceae have been listed in Table 4.

**Table 4.** Comparison of physiological characteristics of *Licheniasvalbardensis* and other members of Kriegeriaceae and Camptobasidiaceae.

Characteristics	1	2	3	4	5	6	7	8	9	10	11	12	13
**Carbon source**													
L-Sorbose	–	d	d, w	w	–	–	–	–	–	–	–	–	+
D-Galactose	+	+	+	–	–	–	–	–	–	–	–	+	d
D-Glucosamine	d	–	–	–	–	–	–	w	d, w	–	–	–	d,w
D-Ribose	+	+	–	+	–	–	–	–	–	–	–	–	–
D-Xylos	d	+	+	w	n/a	n/a	–	w	–	v	–	v	d,w
L-Arabinose	+	+	+	w	+	–	–	–	–	–	–	–	–
L-Rhamnose	+	+	–	w	+	+	–	+	–	–	–	–	–
Sucrose	–	+	+	+	+	+	+	+	+	+	+	v	+
Cellobiose	+	+	–	+	–	–	–	+	–	+	–	–	w
Melibiose	+	d	–	–	–	–	–	–	–	v	–	–	–
Melezitose	+	+	+	+	n/a	–	+	+	+	+	+	–	+
Lactose	+	–	–	–	–	–	–	–	–	v	–	–	–
Raffinose	+	–	–	–	+	+	+	–	–	d	–	–	+
Glycerol	–	+	+	+	–	–	–	w	w	–	+	w	–
myo-Inositol	–	–	–	–	–	–	–	+	–	v	–	–	–
DL-Lactat	w	d	–	+	–	–	–	d	–	n/a	–	n/a	n/a
Citrate	w	+	w	–	–	–	–	d	–	n/a	–	n/a	n/a
**Nitrogen source**													
Nitrite	+	+	–	–	–	–	–	+	–	–	+	+	+
Nitrate	d	+	–	–	+	+	+	+	–	+	+	+	+
Ethylamine	d	+	+	+	+	+	+	+	+	n/a	–	n/a	n/a
**Others**													
Existence of dimorphic stage	+	+	–	–	–	–	–	+	–	–	+	+	+
w/o vitamins	+	+	+	+	n/a	n/a	n/a	–	+	n/a	+	n/a	n/a

1. *Licheniasvalbardensis*; 2. *Kriegeriaeriophori*; 3. *Libkindiamasarykiana*; 4. *Meredithblackwelliaeburnea*; 5. *Phenoliferiaglacialis*; 6. *Phenoliferiapsychrophenolica*; 7. *Phenoliferiapsychrophila*; 8. *Yamadamycesrosulatus*; 9. *Yamadamycesterricola*; 10. *Camptobasidiumarcticum*; 11. *Cryolevoniaschafbergensis*; 12. *Glaciozymaantarctica*; 13. *Psychromycesglacialis.* +. positive; –. negative; d. delayed; w. weak; v. variable (–/+/w/d); n/a = data not available.

**Figure 3.**
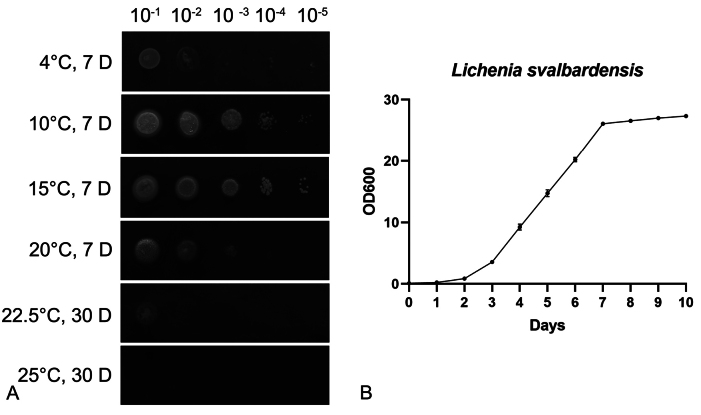
Growth of *Licheniasvalbardensis* at different temperatures **A** cell cultures spotted onto PDA medium and incubated at 4 °C, 10 °C, 15 °C, 20 °C, 22.5 °C, and 25 °C **B** growth curve of *Licheniasvalbardensis* in PBD at 10 °C.

Through examining the effect of temperature on *L.svalbardensis*, we found that this species can grow well from 4 °C to 20 °C (Fig. 3A). The fastest growth rates were observed at 10 °C and 15 °C. However, *L.svalbardensis* remained at no growth at 25 °C or higher temperatures after one month (Fig. 3A). Because pseudohyphae and hyphae were observed for a large proportion at 15 °C and 20 °C, which can influence the values for optical density. The growth curve of *L.svalbardensis* was measured at 10 °C. This species grows slowly and reaches a plateau at 7 days (Fig. 3B).

## ﻿Discussion

The present study reports a new psychrophilic yeast in the Kriegeriaceae family associated with lichen in the Arctic. The isolates in this study were identified as a new genus with *Licheniasvalbardensis* as the type species. It grows fastest at 10 °C and 15 °C. Moreover, pseudohyphae and hyphae can be observed from 10 °C to 20 °C.

Based on modern taxonomic concepts, we propose the isolate as a new genus in Kriegeriaceae. The taxonomic thresholds predicted for yeast species delimitation at the genus level were 96.31% for ITS and 97.11% for LSU recommended by Vu et al. (2016). Phylogenetically, the identity rates of ITS and LSU between *Lichenia* and other genera in Kriegeriaceae are lower than the genera thresholds (Table 3). Although *L.svalbardensis* appeared to be closely related to *Kriegeriaeriophori* in the phylogenetic trees of seven loci (ITS, LSU, SSU, *rpb1*, *rpb2*, *tef1-α*, and *cytb*) and two loci (ITS and LSU) combined (Fig. 1, Suppl. material 1), the identity rates of ITS (88.36% vs. 96.31%) and LSU (95.87% vs. 97.11%) between *Lichenia* and *Kriegeria* are much lower than the genera thresholds (Table 3), especially ITS. Morphologically, the yeast cells of *L.svalbardensis* (9.5–15.6 × 3.4–4.5 µm) are much shorter, significantly different from *K.eriophori* (23.0–29.0 × 4.0–5.0 µm) (Doubles and McLaughlin 1992). Moreover, *L.svalbardensis* is different from *K.eriophori* by host association and sampling location (lichen in the Arctic vs. *Scirpusatrovirens* in North America). Therefore, *Lichenia* was considered a new genus.

The phylogram of two ribosomal loci (ITS and LSU) is similar to the seven loci (ITS, LSU, SSU, *rpb1*, *rpb2*, *tef1-α*, and *cytb*). All of the species in Kriegeriaceaea clustered together with high support values of ML/BI = 94/1.00 in the phylogenetic analyses of seven loci (ITS, LSU, SSU, *rpb1*, *rpb2*, *tef1-α*, and *cytb*). When contaminant genes are deleted from the dataset, *Lichenia* clusters with *Kriegeria*, *Meredithblackwellia*, and *Yamadamyces* in the two phylogenetic trees. However, the *Libkindiamasarykiana* clustered with *Kriegeriopsislivingstonensis* in the phylogram of ITS and LSU, different from the phylogram of seven genes. This may be due to the influence of the missing SSU locus. For example, only ITS and LSU loci were available for *Kriegeriopsislivingstonensis*, which was obtained from lichenicolous specimens instead of cultures (Diederich et al. 2022). Additionally, the low support values between *Libkindiamasarykiana* and *Kriegeriopsislivingstonensis* (ML/BI = 48/0.79) also lead to this result. Therefore, a more robust and complete molecular dataset is needed.

With only ribosomal loci (ITS, LSU, and SSU) incorporated in the analyses, Camptobasidiaceae and Kriegeriaceaea clustered as sisters in the phylogenetic tree (Toome et al. 2013). But when seven loci were used in the phylogenetic analyses, the two families clustered in different clades (Schoutteten et al. 2023). The physiological characters of the two families also showed no obvious association (Table 4). *Licheniasvalbardensis* in this study and other four species in Kriegeriaceaea (*Phenoliferiaglacialis*, *P.psychrophenolica*, and *P.psychrophila*) were confirmed as psychrophilic yeasts (Margesin et al. 2007). Camptobasidiaceae mainly comprises psychrophilic yeasts (De Garcia et al. 2020; Pontes et al. 2020; Perini et al. 2021). Psychrophilia of these species in the two families indicates they may have a close genetic relationship. Due to the lack of more samples and other evidence, the relationship between Camptobasidiaceae and Kriegeriaceaea, as well as the higher systematics of Microbotryomycetes in general, need further study.

The physiological characteristics of all species in Kriegeriaceae show that lactose is assimilated as the sole carbon source and that sucrose is not assimilated for *L.svalbardensis*, which are different from other species in Kriegeriaceae (Table 4). Hence, *L.svalbardensis* can be distinguished from other species in Kriegeriaceae by its capacity to assimilate lactose and sucrose. Moreover, the result of the diazonium blue B reaction and urease activity are positive, agreeing with the characters of Basidiomycetous (Hagler and Ahaearn 1981).

Microorganisms that show no growth above 20 °C can be classified as psychrophiles (Margesin et al. 2003). Colonies of *L.svalbardensis* in the current study grew from 4 °C to 20 °C but not at 25 °C or higher temperatures after one month of incubation (Fig. 3A). Compared to colonies at 4–20 °C after one week of incubation, the colony grows at a significantly lower level at 22.5 °C after one month of incubation (Fig. 3A). Therefore, *L.svalbardensis* could be classified as a psychrophile, which may be due to *L.svalbardensis* being isolated from the polar region. Psychrophilic yeasts with various extracellular enzymatic activities (extracellular amylolytic, proteolytic, lipolytic, esterasic, pectinolytic, chitinolytic, and cellulolytic activities) were screened by Brizzio et al. (2007). These psychrophilic yeasts could be considered a potential source of industrially relevant cold-active enzymes. This implies that *L.svalbardensis* may also become a resource in cold-active industries.

One of the most prominent traits documented for yeasts is their ability to grow in different forms (e.g., *Paracoccidioidesbrasiliensis* and *Yarrowialipolytica*) (Klein and Tebbets 2007; Wu et al. 2020). The morphology of yeasts can be regulated by various environmental factors (Wang et al. 2020). In this study, *L.svalbardensis* can undergo morphological changes between yeast, pseudohyphal, and hyphal forms of growth in different temperatures. Dimorphic switching is a specialized adaptation to the environment (Boyce and Andrianopoulos 2015). *Licheniasvalbardensis* was isolated from lichen. In the collaboration of photosynthetic alga or cyanobacterium, yeast can offer protection from the environment (Spribille et al. 2016). Morphological transformation of this species may be to adapt to different environments, which may contribute to lichen adapting to different temperatures. Although there is not enough evidence that *L.svalbardensis* can offer protection, physiological characteristics (cellobiose, ethanol, melezitose, and melibiose are assimilated as sole carbon sources) imply that *L.svalbardensis* may be symbiotic with photosynthetic species.

## Supplementary Material

XML Treatment for Lichenia

XML Treatment for Lichenia svalbardensis

## ﻿Additional information

### Author ORCIDs

Yukun Bai https://orcid.org/0000-0003-4433-2931

### Data availability

All of the data that support the findings of this study are available in the main text or Supplementary Information.

## Supplementary materials

Supplementary material 1 Phylogram of Microbotryomycetes resulting from a maximum likelihood analysis based on a combined matrix of ITS and LSU Data type tif Explanation note Numbers above the branches indicate ML bootstraps (left, ML BS ≥ 70%) and Bayesian Posterior Probabilities (right, BPP ≥ 0.95). The tree is rooted with *Pseudomicrostromaphylloplana*CBS 8073 and *Ustilagomaydis*CBS 504.76. Isolates from present study are marked in blue and holotype isolates are made in bold. https://binary.pensoft.net/file/1261214This dataset is made available under the Open Database License (http://opendatacommons.org/licenses/odbl/1.0/). The Open Database License (ODbL) is a license agreement intended to allow users to freely share, modify, and use this Dataset while maintaining this same freedom for others, provided that the original source and author(s) are credited.AuthorsNames
