# Supplementary figures and images for: ﻿A new psychrophilic yeast of Kriegeriaceae (Kriegeriales) isolated from lichen in the Arctic, with the description of Licheniasvalbardensis gen. et sp. nov
Source: MycoKeys. 2025 Feb 24;114:95–113. doi: 10.3897/mycokeys.114.135299 (PMC11876982; doi:10.3897/mycokeys.114.135299)

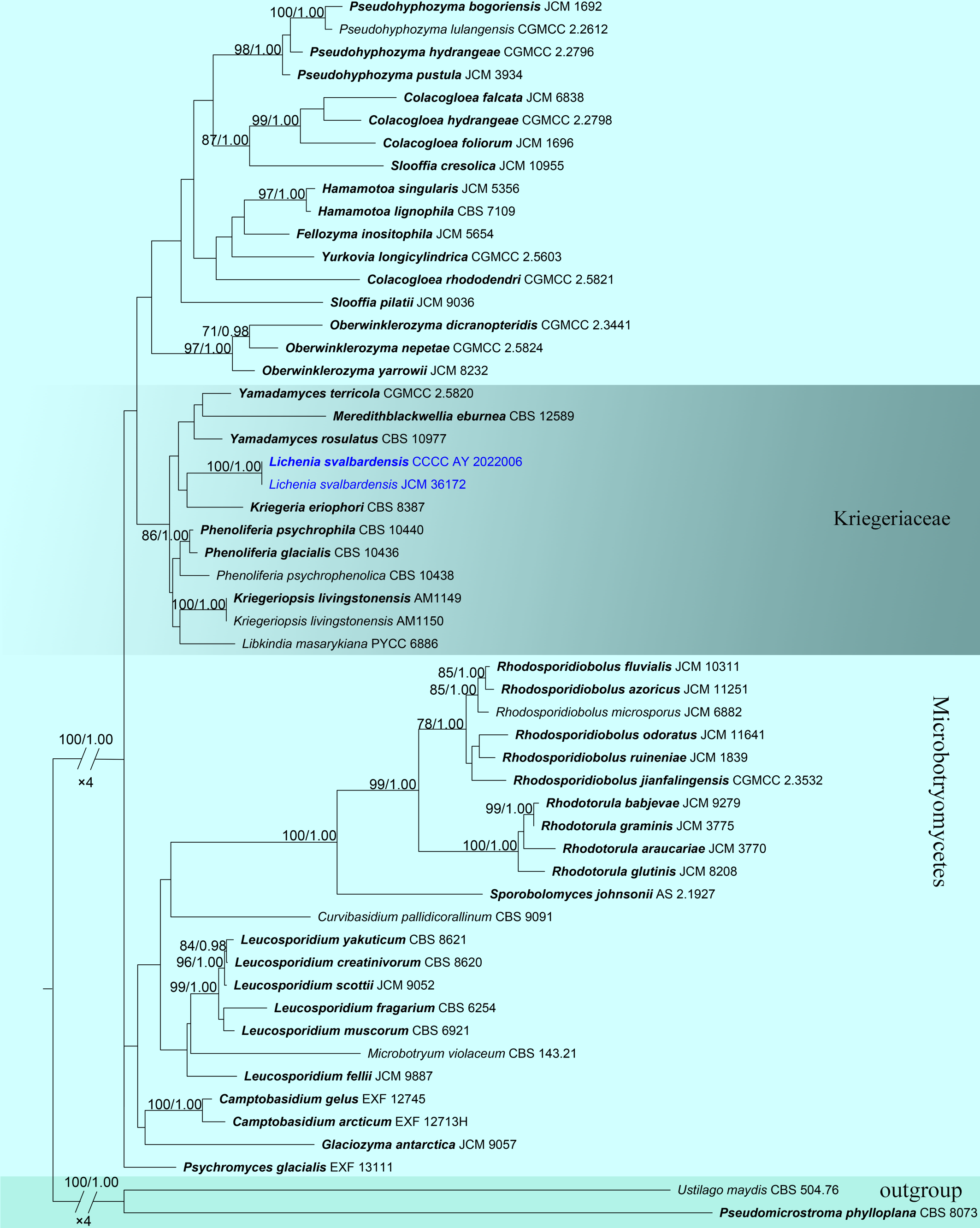

Supplement: Supplementary material 1 — Phylogram of Microbotryomycetes resulting from a maximum likelihood analysis based on a combined matrix of ITS and LSU [file mycokeys-114-095-s001.tif]
